# Critical illness induces alternative activation of M2 macrophages in adipose tissue

**DOI:** 10.1186/cc10503

**Published:** 2011-10-21

**Authors:** Lies Langouche, Mirna B Marques, Catherine Ingels, Jan Gunst, Sarah Derde, Sarah Vander Perre, André D'Hoore, Greet Van den Berghe

**Affiliations:** 1 Laboratory and Department of Intensive Care Medicine, K.U.Leuven, Herestraat 49, 3000 Leuven, Belgium; 2 Department of Abdominal Surgery, K.U.Leuven, Herestraat 49, 3000 Leuven, Belgium

## Abstract

**Introduction:**

We recently reported macrophage accumulation in adipose tissue of critically ill patients. Classically activated macrophage accumulation in adipose tissue is a known feature of obesity, where it is linked with increasing insulin resistance. However, the characteristics of adipose tissue macrophage accumulation in critical illness remain unknown.

**Methods:**

We studied macrophage markers with immunostaining and gene expression in visceral and subcutaneous adipose tissue from healthy control subjects (*n *= 20) and non-surviving prolonged critically ill patients (*n *= 61). For comparison, also subcutaneous *in vivo *adipose tissue biopsies were studied from 15 prolonged critically ill patients.

**Results:**

Subcutaneous and visceral adipose tissue biopsies from non-surviving prolonged critically ill patients displayed a large increase in macrophage staining. This staining corresponded with elevated gene expression of "alternatively activated" M2 macrophage markers arginase-1, IL-10 and CD163 and low levels of the "classically activated" M1 macrophage markers tumor necrosis factor (TNF)-α and inducible nitric-oxide synthase (iNOS). Immunostaining for CD163 confirmed positive M2 macrophage staining in both visceral and subcutaneous adipose tissue biopsies from critically ill patients. Surprisingly, circulating levels and tissue gene expression of the alternative M2 activators IL-4 and IL-13 were low and not different from controls. In contrast, adipose tissue protein levels of peroxisome proliferator-activated receptor-γ (PPARγ), a nuclear receptor required for M2 differentiation and acting downstream of IL-4, was markedly elevated in illness. In subcutaneous abdominal adipose tissue biopsies from surviving critically ill patients, we could confirm positive macrophage staining with CD68 and CD163. We also could confirm elevated arginase-1 gene expression and elevated PPARγ protein levels.

**Conclusions:**

Unlike obesity, critical illness evokes adipose tissue accumulation of alternatively activated M2 macrophages, which have local anti-inflammatory and insulin sensitizing features. This M2 macrophage accumulation may contribute to the previously observed protective metabolic activity of adipose tissue during critical illness.

## Introduction

In our previous study on the role of adipose tissue during critical illness, we reported a remarkable accumulation of macrophages in adipose tissue of prolonged critical ill patients and prolonged critically ill rabbits [1]. Such sudden accumulation of adipose tissue macrophages is a known feature of obesity, where it is linked with increased insulin resistance [2,3]. Recent findings demonstrate that adipose tissue of healthy, lean individuals contains a resident population of "alternatively activated" or M2 macrophages, whereas obesity related adipose tissue expansion appears to induce an infiltration of "classically activated" or M1 macrophages [4,5]. These M1 macrophages produce pro-inflammatory cytokines, such as tumor necrosis factor alpha (TNF-α), thereby contributing to a local and systemic chronic inflammatory status and insulin resistance [2-5]. In obesity, the increase in adipocyte cell size is suggested to be the trigger for this macrophage accumulation, as there is a strong positive correlation between adipocyte cell size and the amount of macrophages [2,3].

Why critical illness appears to be associated with increased infiltration of macrophages in adipose tissue is not clear. In contrast with obesity, critical illness induced a decrease in adipocyte cell size [1]. Furthermore, whether critical illness induces an increase in M1 or M2 macrophages in adipose tissue remains to be identified. M1 macrophages are mainly activated by interferon-gamma (IFN-γ). They have an enhanced pro-inflammatory cytokine production (TNF-α, IL-6, IL-12) and generate excessive nitric oxide levels through inducible nitric-oxide synthase (iNOS) activation. In contrast, macrophages are polarized to the M2 state by IL-4 and IL-13. M2 macrophages secrete high levels of the anti-inflammatory cytokine IL-10 and produce arginase-1, which metabolizes arginine to ornithine. By directly competing for the substrate arginine, activation of arginase-1 will also antagonize iNOS action [6,7].

We previously reported that adipose tissue from critically ill patients displayed a larger number of small adipocytes in response to critical illness, with an increased ability to take up circulating glucose and triglycerides [1]. These changes rather point to increased insulin sensitivity in the adipose tissue and would contradict with a local increase of pro-inflammatory, TNF-α producing M1 macrophages. Therefore, we hypothesized that critical illness induces an accumulation of anti-inflammatory, insulin sensitizing M2 macrophages. To determine the characteristics of the macrophage accumulation in adipose tissue of the critically ill, we studied abdominal subcutaneous and visceral adipose tissue biopsies and serum of 61 non-surviving prolonged (more than three days) critically ill patients and 20 demographically matched controls. Finally, we confirmed our findings in 15 *in vivo *sampled abdominal adipose tissue needle biopsies from prolonged critically ill patients.

## Materials and methods

### Patients

Postmortem biopsy samples of abdominal subcutaneous and omental adipose tissue were harvested within minutes after death from 61 long-stay critically ill patients with an intensive care unit (ICU) stay of at least three days. Patients were all enrolled in a large prospective, randomized controlled study on the effects of intensive insulin therapy on outcome of critical illness [8]. The detailed protocol of the study has been previously published [8]. We collected biopsies from 33 conventionally treated patients and 28 samples from intensive insulin treated patients. For postmortem tissue sampling for academic purposes, each patient or his/her legal representative consented upon admission, via a hospital-wide information and consent procedure, which requires active opting-out when not consenting. Opting-out remained possible until time of death. This strategy was approved by the Institutional Ethical Review Board (ML1820). The baseline and outcome characteristics of the critically ill patients from whom postmortem adipose tissue biopsies were collected, are described in Table 1. For comparison, we also collected abdominal subcutaneous and omental adipose tissue biopsy samples from demographically matched patients, who were not critically ill, and who underwent elective abdominal surgery for restorative rectal resection (ML 2707). These patients provided written informed consent prior to the procedure. From 15 long-stay patients, we collected *in vivo *abdominal subcutaneous adipose tissue needle biopsies on Day 7 of their ICU stay. The *in vivo *biopsies were taken after a specific written informed consent from the patient or his/her legal representative (ML 4190). The baseline characteristics of the critically ill patients from whom *in vivo *adipose tissue biopsies were collected are described in Table 2. The protocols and all consent forms were approved by the Institutional Ethical Review Board (ML 1820 and ML 2707, ML 4190). All tissue samples were snap-frozen in liquid nitrogen and stored at -80°C until analysis.

**Table 1:** Characteristics of healthy controls and non surviving critically ill patients

	Healthy controls	Critically ill patients: *postmortem *biopsies
Number	20	61
Gender (no. male)	14	36
Age (yr) (mean ± SD)	70 ± 12	67 ± 15
BMI (kg/m^2^) (mean ± SD)	25.0 ± 2.6	24.1 ± 3.5
History of diabetes (no.)	3	9
APACHE-II score on admission (median (IQR))	-	26 (20 to 34)
Admission diagnose	-	
- *Cardiovascular*		5
- *Gastrointestinal or liver*		5
- *Hematologic or oncologic*		10
- *Neurologic*		3
- *Renal*		2
- *Respiratory*		30
- *Other*		6
ICU stay (median (IQR))	0	10 (6 to 19)
Cause of death in ICU	-	
- *Severe brain damage*		2
- *Respiratory failure*		26
- *Therapy resistant septic shock/cardiovascular collapse*		14
- *Persistent MOF after septic or SIRS induced shock*		19

APACHE II, Acute Physiology and Chronic Health Evaluation II; BMI, body mass index; ICU, intensive care unit; IQR, interquartile range; MOF, multiple organ failure; SD, standard deviation; SIRS, systemic inflammatory response syndrome; Yrs, years.

**Table 2:** Characteristics of surviving critically ill patients

Number	15
Gender (no. male)	11
Age (yr) (mean ± SD)	56 ± 16
BMI (kg/m^2^) (mean ± SD)	28.1 ± 5.2
APACHE-II score on admission (median (IQR))	30 (23 to 40)
Admission diagnose	
- *Cardiac surgery*	1
- *Transplantation*	1
- *Trauma, burns or reconstructive surgery*	5
- *Complicated thoracic surgery*	2
- *Complicated vascular surgery*	1
- *Gastroenterologic or hepatic disease*	1
- *Complicated neurosurgery*	2
- *Hematological or oncological disease*	1
- *Renal disease*	1
ICU stay (median (IQR))	22 (13 to 28)
Deaths during intensive care - no. (%)	1 (7%)

The *in vivo *abdominal subcutaneous adipose tissue biopsies were sampled at Day 7 of the patients ICU stay. APACHE II, Acute Physiology and Chronic Health Evaluation II; BMI, body mass index; ICU, intensive care unit; IQR, interquartile range; MOF, multiple organ failure; SD, standard deviation; SIRS, systemic inflammatory response syndrome; Yrs, years.

### Adipocyte morphology

Tissue samples were fixed in 6% paraformaldehyde overnight. Paraffin wax sections of 6 μm were processed for immunostaining. Sections were stained with a primary anti-macrophage CD68 antibody (Dako, Glostrup, Denmark) and CD163 antibody (Santa Cruz Biotechnology, Santa Cruz, CA, USA) and counterstained with hematoxylin. Staining was evaluated at a 10 × and 40 × magnification using a Leica DM3000 Microscope (Leica, Wetzlar, Germany).

### Gene expression

Tissue samples were homogenized with a Precellys 24 machine using CK14 tubes (Bertin Technologies, Villeurbanne, France) containing ceramic beads at 6,500 rpm for one cycle of 45 sec in Qiazol (Qiagen, Hilden, Germany). RNA was isolated using the RNeasy mini RNA isolation kit (Qiagen) and quantified by Nanodrop spectrophotometer (ND-1000, Nanodrop Technologies, Wilmington, DE, USA). Samples were treated with DNAse to remove all contaminating genomic DNA. A total of 500 to 250 ng of total RNA was reverse-transcribed using Superscript III Reverse Transcriptase (Invitrogen, Merelbeke, Belgium) and random primers (Invitrogen). In a first run, all postmortem samples and healthy controls were reverse transcribed simultaneously. In a second run, all *in vivo *samples and healthy controls were reverse transcribed simultaneously. One-fifth of dilutions of reverse transcribed RNA samples were quantified in real time with the StepOne Plus (Applied Biosystems, Carlsbad, CA, USA), which uses TaqMan chemistry for highly accurate quantization of mRNA levels. Unknown samples were run in duplicate and individual samples with a Cycle threshold (Ct) value standard deviation greater than 0.3 were reanalyzed. Data were analyzed using the comparative Ct method. Data are expressed normalized to glyceraldehyde-3-phosphate dehydrogenase (GAPDH) expression and as a fold change of the mean of the controls. GAPDH, arginase-1, IL-10, iNOS, CD163, IL-4, IL-13 and TNF-α gene expression assays from Applied Biosystems were used.

### Serum analyses

Serum levels of IFN-γ, IL-4, IL-10 and IL-13 were measured with high sensitivity ELISAs (eBioscience, San Diego, CA, USA) according to the manufacturer's protocol.

### Immunoblot analysis

Tissue samples were weighed and homogenized with a Precellys 24 machine using CK14 tubes containing ceramic beads at 6,000 rpm for one cycle of 30 sec in lysis buffer containing phosphatase inhibitors. The homogenates were centrifuged for 10 minutes at 5,000 rpm and 4°C. The protein content in the homogenate was determined with a Coomassie Protein Assay Reagent (Thermo Scientific Pierce, Rockford, IL, USA). An equal amount of protein was loaded for each sample and homogenates were separated by denaturating SDS-PAGE and immunoblotted with a specific Ab against peroxisome proliferator-activated receptor-γ (PPARγ) (ABCAM, Cambridge, UK), and with a species-specific HRP-conjugated secondary Ab. All blots were analyzed using Image Master Software (GE Healthcare Europe, Chalfont St. Giles, UK).

### Statistics

We used ANOVA and unpaired t-test for normally distributed data and the non-parametric Kruskal-Wallis and Mann-Whitney *U *test when data appeared to be not normally distributed. Statistical significance was considered when *P*-values were ≤ 0.05.

## Results

### Characterization of the macrophages

**Figure 1:**
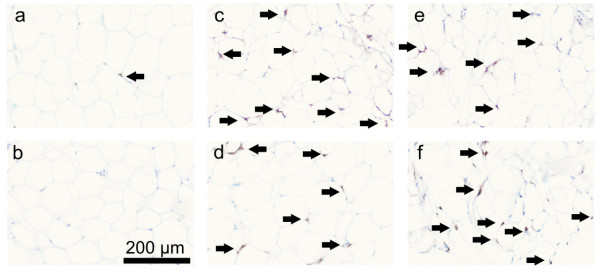
**Macrophage staining with CD68 in abdominal subcutaneous adipose tissue**. Biopsies from two non-critically ill volunteers (a, b) and four critically ill, non-surviving patients (c-f).

We described earlier that subcutaneous and visceral adipose tissue biopsies from critically ill patients display a large number of CD68 positive cells, which is a general macrophage marker. This was in sharp contrast with biopsies from healthy controls where only occasionally some CD68 positive cells were observed [1]. Positive cells displayed macrophage morphology and were found scattered throughout the adipose tissue (Figure 1).

**Figure 2:**
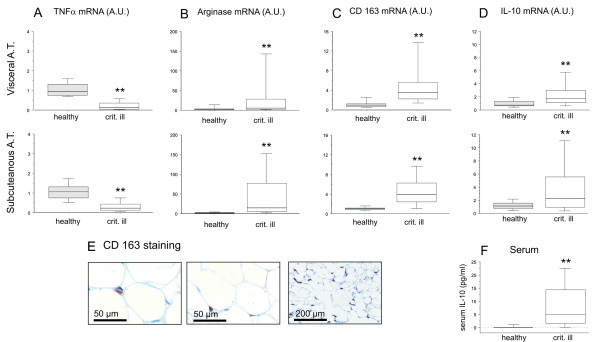
**M1 and M2 macrophage markers in adipose tissue from healthy controls and critically ill, non-surviving patients**. Adipose tissue (A.T.) mRNA levels of (**A**) TNF-α, (**B**) Arginase-1, (**C**) CD163, (**D**) and IL-10 (D). (**E**) Adipose tissue biopsy staining for CD163 from one representative patient, and (**F**) serum IL-10 levels on the day of sampling. Databoxes present median and interquartile range; whiskers represent the 10^th ^and the 90^th ^percentiles. Grey boxes represent healthy controls undergoing elective surgery (healthy, *n *= 20); white boxes are critically ill patients (crit. Ill, *n *= 61). Different from healthy controls with *P ≤ 0.05, ** *P *≤ 0.01. AU, arbitrary units.

Depending on the activation, macrophages can polarize to a M1- or M2- phenotype. Each phenotype expresses specific markers, allowing the identification of the phenotype. TNF-α and iNOS are specific markers for the M1 phenotype. In a selection of 61 human postmortem adipose tissue biopsies, TNF-α gene expression was decreased in both the subcutaneous and visceral depot, compared to healthy controls (Figure 2A). We could also demonstrate a decreased iNOS gene expression in visceral adipose tissue (from 0.81 (0.54 to 1.3) in healthy individuals to 0.34 (0.23 to 0.81) median (IQR) in non-surviving critically ill patients, *P *= 0.0095), with unaltered levels in the subcutaneous adipose tissue of nonsurviving critically ill patients (from 0.91 (0.70 to 1.22) in health, to 0.82 (0.31 to 2.05) in critical illness, *P *= 0.9). These observations suggest that the macrophages we identified in adipose tissue of critically ill patients are not of the pro-inflammatory M1-type.

Therefore, we tested adipose tissue expression level of the specific M2 marker arginase-1 in the same selection of 61 nonsurviving critically ill patients. In contrast with the M1 markers, arginase-1 gene expression was increased several-fold in both visceral and subcutaneous depots (Figure 2B). Two other markers for M2 phenotype macrophages, CD163 and IL-10, were comparably elevated in the human postmortem visceral and subcutaneous adipose tissue biopsies (Figure 2C, D). Because gene expression levels were measured in total adipose tissue, we confirmed the cell specificity of the M2-markers with immunostaining in a random selection of the available postmortem biopsies. In the nonsurviving critically ill patients, 9 out of 10 omental adipose tissue biopsies and 10 out of 10 subcutaneous adipose tissue biopsies stained positive for CD163 whereas in healthy controls none of the tested omental (*n *= 10) nor subcutaneous (*n *= 10) biopsies stained positive for CD163. Positive CD163 staining pattern in the biopsies from critically ill patients was comparable to the CD68 staining pattern (Figure 2E).

Functionally, M2 macrophages will produce high levels of IL-10, whereas iNOS expression and concomitant NO-production might be reduced. Indeed, in serum samples taken on the day of the biopsy, IL-10 was clearly elevated in the patients compared to healthy controls (Figure 2F). However, the levels did not correlate significantly with tissue gene expression level, pointing to other IL-10 sources besides adipose tissue residing macrophages.

### Possible triggers for macrophage accumulation

The primary trigger for M1 type activation is IFN-γ. In serum samples collected on the day of the biopsy, IFN-γ levels were low (mean ± SE 1.77 ± 0.49 pg/ml) in critically ill patients, but not different from healthy control samples (*P *= 0.9). The most common activators of M2 macrophages are the anti-inflammatory cytokines IL-4 and IL-13. IL-4 serum levels were however below detection limit (90% of the patients) or very low. IL-13 levels were low (6.24 ± 2.70 pg/ml), and not different from healthy control samples (*P *= 0.9). Adipocytes and residing macrophages can also locally produce cytokines, which might trigger M2 type activation. Gene expression of IL-4 and of IL-13 was low to undetectable in both visceral and omental adipose tissue postmortem biopsies and no difference could be detected from healthy volunteers.

**Figure 3:**
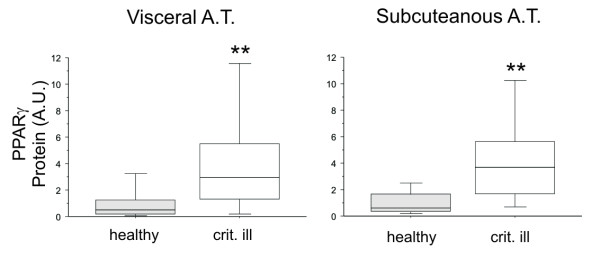
**Adipose tissue levels of protein PPARγ from healthy controls and critically ill, non-surviving patients**. Databoxes present median and interquartile range; whiskers represent the 10^th ^and the 90^th ^percentiles. Grey boxes represent healthy controls undergoing elective surgery (healthy, *n *= 20); white boxes are critically ill patients (crit. Ill, *n *= 61). Different from healthy controls with ** *P *≤ 0.01. AT, adipose tissue; AU, arbitrary units.

More downstream of cytokine activation, PPARγ, which is a nuclear receptor expressed in high levels in adipose tissue, is a possible trigger for M2 polarization. It plays a pivotal part in adipogenesis, lipid biosynthesis, insulin sensitivity, but has also been demonstrated to stimulate the formation of M2 macrophages. IL-4 and IL-13 are known to activate PPARγ. With Western blotting, we could clearly demonstrate increased levels of PPARγ in both subcutaneous and visceral postmortem adipose tissue biopsies from critically ill patients, compared to healthy controls (Figure 3).

### Confirmation in vivo

We were able to collect *in vivo *small needle biopsies from abdominal subcutaneous adipose tissue in 15 critically ill patients at Day 7 of their ICU stay. Of the 15 patients from which we sampled *in vivo *biopsies, 14 patients survived their ICU and hospital stay. Only one patient died in the ICU, and this was after 121 days (114 days after the day of the biopsy).

**Figure 4:**
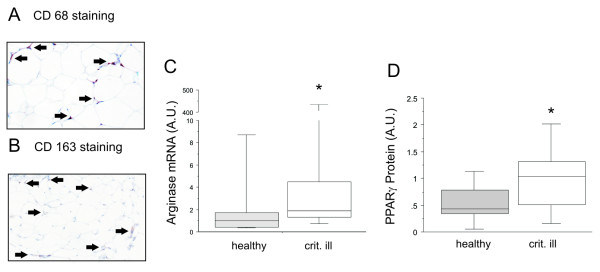
**Macrophage markers in adipose tissue from (healthy controls and critically ill, surviving patients**. Positive CD68 (**A**) and CD163 (**B**) staining in *in vivo *abdominal subcutaneous biopsies from critically ill patients. (**C**) Adipose tissue levels of arginase-1 gene expression. (**D**) Adipose tissue levels of protein PPARγ. Databoxes present median and interquartile range; whiskers represent the 10^th ^and the 90^th ^percentiles. Grey boxes represent healthy controls undergoing elective surgery (healthy, *n *= 20); white boxes are critically ill patients from which *in vivo *biopsies were collected (crit. Ill, *n *= 15). Different from healthy controls with * *P *≤ 0.05. AU, arbitrary units.

Paraffin sections of the *in vivo *biopsies stained positive for CD68 (Figure 4A) and CD163 (Figure 4B), confirming our findings of the postmortem biopsies. We also quantified arginase-1 and TNF-α gene expression in the *in vivo *biopsies together with healthy control samples. Confirming our findings in the non-surviving patients, we could demonstrate in these surviving patients a clearly increased gene expression of the M2-marker arginase-1 (Figure 4C), whereas TNF-α was not altered compared to healthy controls (healthy controls 0.98 (0.75 to 1.37), critically ill patients 0.90 (0.35 to 1.83), *P *= 0.37). Samples were also used for protein analysis, with which we could confirm an increase in PPARγ compared to healthy controls (Figure 4D).

### Impact of insulin or steroid treatment

Treatment with insulin or corticosteroids might influence the inflammatory state of the patient and consequently influence macrophage polarization. The patients from which we studied postmortem biopsies were all enrolled in a randomized controlled trial on blood glucose control [8]. From the 61 studied nonsurviving patients, 33 patients were on conventional insulin regimen, which resulted in mean blood glucose levels of 157 ± 4 mg/dl (mean ± SE), 28 patients were on intensive insulin therapy which resulted in mean blood glucose levels of 110 ± 3 mg/dl. Both patient groups displayed elevated insulin levels on the day of biopsy, 12.1 (7.0 to 31.2) mIU/l (median (IQR)) for the conventional group, 29.3 (10.2 to 83.2) mIU/l for the intensive insulin treated group, and only 6.2 (6.0 to 7.4) mIU/l for the healthy control group. Although the two treatment groups displayed different glucose levels and different circulating insulin levels, this did not result in a different expression level of any of the tested macrophage markers. Also, the tested circulating cytokines did not differ between the two treatment groups. Although insulin is known for its anti-inflammatory actions [9,10], insulin therapy had apparently no impact on either macrophage marker expression profile or cytokine levels. This confirms earlier findings in which insulin therapy had no [11] or very minor [12] impact on circulating cytokines, irrespective of its anti-inflammatory actions [10].

In the postmortem patient population, 51 out of 61 patients (84%) received steroids during their ICU stay. In the *in vivo *patient population, 6 out of 15 patients (40%) received steroids before the biopsy sampling. Treated and untreated patients displayed similar levels for the tested macrophage markers and circulating cytokines. Treatment with steroids thus appeared not to affect macrophage polarization in adipose tissue.

## Discussion

In this study, we demonstrated that critical illness induces an accumulation of alternatively active M2 macrophages in adipose tissue. High levels of PPARγ in the adipose tissue of critically ill patients could explain this accumulation, rather than high circulating cytokine levels.

The activation of M1 macrophages is a classical feature of cellular immunity to infection while the alternative activation of M2 macrophages is considered to have a role in humoral immunity and repair. Alternatively activated M2 macrophages have been implicated in immunity, inflammation, allergy, parasitic infections, repair, metabolic functions and malignancy [6]. Alternatively activated macrophages express high levels of the enzyme arginase-1 [13]. Arginase-1 was classically an enzyme considered to be exclusively expressed in the liver, but is now recognized to also occur in cells of the immune system. Arginase-1 is the enzyme that produces ornithine, the precursor of polyamines and proline. Polyamines participate in a variety of cellular processes, such as cell proliferation and tissue regeneration [14]. Activation of arginase-1 also counteracts iNOS action by competing for the same substrate arginine, thereby inhibiting the biosynthesis of nitric oxide [15]. CD163 and IL-10, two other markers of alternative macrophage activation, were also up-regulated in adipose tissue of critically ill patients [16,17]. CD163 is the scavenging receptor for hemoglobin, exclusively expressed by monocytes and tissue macrophages [17,18]. Macrophages are the primary scavengers of hemoglobin after systemic hemolysis and during wound healing, two processes which are often ongoing during critical illness. IL-10 is a strong anti-inflammatory cytokine that conditions the activation and function of immune cells, selectively blocks the expression of pro-inflammatory genes and enhances the expression of anti-inflammatory molecules [19]. In critically ill patients, adipose tissue IL-10 expression, as well as IL-10 in circulation, was clearly elevated. We could not, however, demonstrate a clear association between circulating IL-10 levels and expression levels in adipose tissue, which suggests other possible sources for IL-10. Possibly, the produced IL-10, but also CD163 and arginase in adipose tissue macrophages only play a paracrine role, thereby preventing adipose tissue injury from excess inflammation and insulin resistance. Remarkably, circulating mononuclear cells from critically ill patients also appear to be switched to the M2-phenotype [20,21].

Once the polarization state of the macrophages was determined, we tried to identify the trigger for the M2 polarization in adipose tissue of critical illness. Macrophages are polarized to the M1 state through the classical activation pathway by the T_H_1 cytokine IFN-γ, while the alternative activation with T_H_2 cytokines IL-4 and IL-13 polarizes macrophages to the M2 phenotype [6,13,22]. In our experimental setup, IFN-γ, IL-4 or IL-13 was not up-regulated in circulation. Also, locally produced cytokines might trigger macrophage polarization. We, however, did not find an up-regulation of IL-4 or IL-13 expression. Our methodology in which we measured gene expression in total adipose tissue might possibly not be sensitive enough to measure a mild up-regulation of IL4 or IL13 only in adipocytes. However, critically ill patients often suffer from systemic inflammatory response syndrome (SIRS) or sepsis, associated with high circulating levels of several pro-inflammatory and anti-inflammatory cytokines. Possibly, it is this cocktail of cytokines, which is responsible for the pro-inflammatory activation of macrophages in adipose tissue.

We were able to identify a more downstream activator of M2 polarization. PPARγ is a transcription factor that has been characterized as a key signal in alternative M2 macrophage activation [13,23]. We clearly found increased PPARγ protein levels in adipose tissue of critically ill patients, both in postmortem biopsies and *in vivo*. Although it has been demonstrated that IL-4 and IL-13 can activate PPARγ, other possible activators for PPARγ are fatty acids and prostaglandines [13,24,25]. Possibly high circulating lipid levels, often observed in critically ill patients, play a role in the observed PPARγ activation [26-28].

The following limitation in the study design should be highlighted. Because the available biopsies were all snap-frozen at the time of biopsy, we were not able to isolate adipocytes from the stromal cell fraction. Gene and protein expression were, therefore, quantified in whole adipose tissue homogenates.

## Conclusions

In conclusion, we demonstrated that unlike obesity, critical illness evokes accumulation of alternatively activated M2 macrophages in adipose tissue. M2 accumulation in the adipose tissue could be considered as a beneficial response promoting anti-inflammatory actions, wound healing and scavenging of hemoglobin. Whether or not the M2 macrophages only play a local role in the adipose tissue, or are a source of polyamines and soluble CD163 in the circulation cannot be concluded from this study. Possibly also other tissues, such as the liver, might display increased macrophage infiltration and M2 polarization during critical illness, for which further study is needed.

## Key messages

• Irrespective of the admission diagnosis, prolonged critically ill patients display accumulation of macrophages in adipose tissue.

• These tissue macrophages display characteristics of alternatively activated M2 macrophages.

• The key trigger for this accumulation might be an increase in PPARγ activity.

## Abbreviations

APACHE II: Acute Physiology and Chronic Health Evaluation II; AT: adipose tissue; AU: arbitrary units; BMI: body mass index; GAPDH: glyceraldehyde-3-phosphate dehydrogenase; IL: interleukin; iNOS: inducible nitric-oxide synthase; IQR: interquartile range; MOF: multiple organ failure; PPARγ: peroxisome proliferator-activated receptor-γ; SD: standard deviation; SE: standard error; SIRS: systemic inflammatory response syndrome; TNF: tumor necrosis factor; Yrs: years.

## Competing interests

The authors declare that they have no competing interests.

## Authors' contributions

LL designed and coordinated the study, participated in the data collection, analyzed the results and drafted the manuscript. MM participated in the design of the study and helped to draft the manuscript. JG, AD and SD participated in the design of the study and the collection of the biopsies. CI participated in the molecular gene expression studies. SVP participated in the molecular gene expression studies and immunostainings. GV participated in the study design and coordination and helped to draft the manuscript. All authors read and approved the final manuscript for publication.
